# Role of Sulfide Quinone Oxidoreductase and Supersulfides in Hepatic Ischemia–Reperfusion Injury in Mice

**DOI:** 10.3390/antiox15010094

**Published:** 2026-01-12

**Authors:** Shinnosuke Takamori, Kazuhiro Shirozu, Eizo Marutani, Tsuyoshi Takata, Yukie Mizuta, Takahito Kawano, Masaharu Murata, Tomoaki Ida, Tetsuro Matsunaga, Takaaki Akaike, Ken Yamaura, Tomohiko Akahoshi

**Affiliations:** 1 Department of Anesthesiology and Critical Care Medicine, Graduate School of Medical Sciences, Kyushu University, Fukuoka 819-0395, Japan; takamori.shinnosuke.955@s.kyushu-u.ac.jp (S.T.); mizuta.yukie.330@m.kyushu-u.ac.jp (Y.M.); yamaura.ken.361@m.kyushu-u.ac.jp (K.Y.); 2 Department of Advanced Emergency and Disaster Medicine, Graduate School of Medical Sciences, Kyushu University, Fukuoka 819-0395, Japan; akahoshi.tomohiko.006@m.kyushu-u.ac.jp; 3 Operating Rooms, Kyushu University Hospital, Fukuoka 812-8582, Japan; 4 Anesthesia Center for Critical Care Research, Department of Anesthesia, Critical Care and Pain Medicine, Massachusetts General Hospital and Harvard Medical School, Boston, MA 02114, USA; 5 Department of Redox Molecular Medicine, Tohoku University Graduate School of Medicine, Sendai 980-8575, Japan; tsuyoshi.takata.a5@tohoku.ac.jp (T.T.); tomoaki.ida.a5@tohoku.ac.jp (T.I.); tetsuro.matsunaga.e5@tohoku.ac.jp (T.M.); takaaki.akaike.b4@tohoku.ac.jp (T.A.); 6 Center for Advanced Medical Innovation, Kyushu University, Fukuoka 812-8582, Japan; t-kawano@camiku.kyushu-u.ac.jp (T.K.); m-murata@camiku.kyushu-u.ac.jp (M.M.); 7 Comprehensive Center for Infectious Disease Control, Graduate School of Medicine, Akita University, Akita 010-8502, Japan; 8 Key Research Laboratory, Akita University, Akita 010-8502, Japan

**Keywords:** sulfur metabolome, hydrogen sulfide, sodium trisulfide, glutathione trisulfide, oxidative stress

## Abstract

Hepatic ischemia–reperfusion injury (IRI) is a critical clinical condition associated with liver transplantation and acute liver injury. This study investigated the role of sulfide quinone oxidoreductase (SQOR) and its downstream product, supersulfides, in hepatic IRI. C57BL/6NJ mice were subjected to 45 min of partial hepatic ischemia, followed by reperfusion lasting 4 h. Control of shRNA mediated knockdown of SQOR expressing adeno-associated viral vectors were administered 3 weeks prior to liver ischemia. In the shRNA-mediated knockdown of SQOR group, the hydro-trisulfide donor sodium trisulfide was administered daily for 1 week prior to the induction of liver ischemia. SQOR played a crucial protective role during hepatic IRI by facilitating electron transport to the mitochondrial respiratory chain and maintaining the oxidized and reduced nicotinamide adenine dinucleotide ratio. Administration of sodium trisulfide, exhibited protective effects against hepatic IRI. Sodium trisulfide restored the oxidized and reduced nicotinamide adenine dinucleotide ratio, reduced oxidative stress, and preserved the expression of key enzymes involved in the sulfide oxidation pathway. SQOR and supersulfides contribute to hepatic protection against IRI, likely through their potent antioxidative and redox-regulating functions, and highlight sodium trisulfide as a potential therapeutic agent.

## 1. Introduction

Acute liver injury (ALI) is caused by various diseases and conditions, such as infection, tumors, sepsis, heart failure, surgical treatment, and dehydration. Hepatic ischemia–reperfusion injury (IRI) is one of the mechanisms underlying ALI and is caused by diseases, conditions, and operations, especially liver transplantation surgery. Liver transplantation is widely used to treat end-stage liver failure and is performed in >30,000 cases annually worldwide [1].

As a critical regulator of liver function, persulfides are critically involved in the etiology of various liver disorders, such as nonalcoholic steatohepatitis, hepatic fibrosis, hepatic IRI, and liver cancer [2]. Hydrogen sulfide is oxidized by sulfide quinone oxidoreductase (SQOR) to generate persulfides, such as cysteine persulfides (CysSSH) and glutathione persulfides (GSSH). These persulfides are subsequently metabolized by downstream sulfur-metabolizing enzymes, including ethylmalonic encephalopathy protein 1 (ETHE1) and thiosulfate sulfurtransferase (TST). Among these, metabolites comprising inorganic and organic sulfur with sulfur catenation are called “supersulfides” [3], which have various effects, including antioxidant properties, anti-inflammatory activities, signaling functions, and mitochondrial activation, and have garnered significant attention in recent years [4,5,6]. Supersulfides are now widely recognized as universal bioactive metabolites that are physiologically synthesized in all organisms and act as electron acceptors produced by the mitochondrial electron respiratory chain [7,8]. Per a previous study, when Na_2_S_3_ was administered to mice in a 1-methyl-4-phenyl-1,2,3,6-tetrahydropyridine-induced Parkinson’s disease model, it may have generated supersulfides and demonstrated neuroprotective effects [9], and adding Na_2_S to culture medium increases intracellular supersulfides levels, leading to nuclear factor erythroid 2-related factor 2 (Nrf2) activation and glutathione (GSH) increase, thereby exhibiting cellular protective effects [10]. Sodium trisulfide (Na_2_S_3_) dissociates in the body, releasing sulfur anions, such as S_3_^2–^, which can interact with thiol-containing molecules, such as cysteine, resulting in supersulfides formation. Recently, glutathione trisulfide (GSSSG) has been identified as an exogenous supersulfides donor. In contrast to Na_2_S_3_, which is known to release H_2_S under acidic conditions, GSSSG directly augments cellular supersulfide levels and exhibits potent antioxidative and cytoprotective activities [9,11,12,13]. GSSSG has also been reported to have potential as a novel therapy for neurodegenerative disorders, including for Parkinson’s disease and for delayed paraplegia following spinal cord ischemia [9,14]. However, the effects of supersulfides on hepatic IRI have not been sufficiently examined.

Persulfides are produced by enzymes, including cystathionine β-synthase (CBS), and cystathionine γ-lyase (CSE) [8,15,16]. SQOR is a key enzyme involved in sulfide metabolism in the mitochondria [17]. The oxidation of sulfide by SQOR is the first step and is considered the rate-limiting step in sulfide oxidation [18]. Recent studies have shown that the sensitivity of the brain to hypoxia is inversely related to the levels of SQOR and the capacity to catabolize sulfide in mice, rats, and naturally hypoxia-tolerant ground squirrels. Therefore, SQOR plays a protective role in hypoxic brain injury [2]. However, the role of SQOR in the liver and hepatic IRI remains unclear.

This study aimed to investigate the role of SQOR in hepatic IRI. The effects of supersulfides produced by SQOR in the context of hepatic IRI were also investigated.

## 2. Materials and Methods

### 2.1. Animals

Male C57BL/6NJ mice (age, 6 or 9 weeks; weight, 18–25 g) were purchased from Charles River Laboratories Japan, Inc. (Yokohama, Japan). The mice were kept in controlled chambers (22 ± 2 °C and 12 h light/dark cycle) and had access to water and food ad libitum for a week in the animal center of Kyushu University. Overall, 36 mice were used for the final analyses in this study. Mice were allocated into six experimental groups: sham, IRI, AAV-control+IRI, AAV-shSQOR+IRI, AAV-shSQOR+Na_2_S_3_+IRI, and AAV-shSQOR+GSSSG+IRI (n = 6 mice per group). Each experiment was conducted using independent cohorts, and mice were not reused across different experimental protocols. Animals used for preliminary or optimization experiments were not included in the final analyses. Animals were included if ischemic reperfusion surgery and tissue collection were completed without technical failure. Animals were excluded only in case of unexpected intraoperative death or technical issues that compromised sample integrity. All surgical procedures were performed under isoflurane anesthesia, and efforts were made to minimize pain and distress. All experiments were conducted according to the ARRIVE guideline 2.0 for animal research.

### 2.2. Hepatic IRI Model

**Figure 1:**
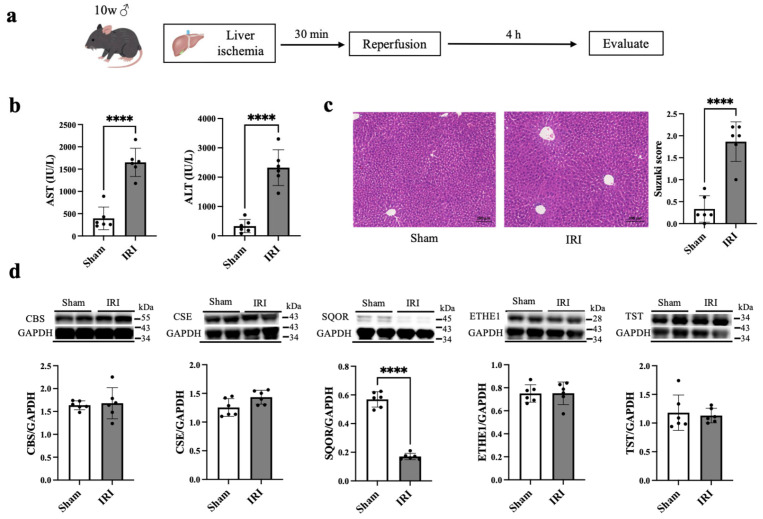
Liver function and histological changes and level of sulfide quinone oxidoreductase (SQOR) in hepatic ischemia–reperfusion injury in mice. (**a**) Flowchart of the experimental protocols. (**b**) The levels of aspartate aminotransferase (AST) and alanine aminotransferase (ALT) 4 h after reperfusion in mice. (**c**) Representative images of hematoxylin and eosin (H&E) staining 4 h after reperfusion in mice. The scale bars are 50 μm in H&E images. (**d**) Upper part of the figure shows representative Western blotting images of cystathionine β-synthase (CBS), cystathionine γ-lyase (CSE), sulfide quinone oxidoreductase (SQOR), ethylmalonic encephalopathy 1 (ETHE1), thiosulfate sulfurtransferase (TST), and glyceraldehyde-3-phosphate dehydrogenase (GAPDH) protein levels in liver extracts from sham and IRI mice. Lower part of the figure shows differences in CBS/GAPDH, CSE/GAPDH, SQOR/GAPDH, ETHE1/GAPDH, and TST/GAPDH in each group. The data are presented as n = 6 per group. Significant differences between groups were determined using one-way analysis of variance. Data are expressed as the means ± standard deviations, **** *p* < 0.0001.

The mice (age, 10 weeks) were randomly allocated to two groups: the sham (n = 6) and IRI (n = 6) groups. The mice were anesthetized with 1–2% isoflurane. In the sham group, the liver was exposed without clamping, and the incision was subsequently sutured. In the IRI group, the portal vein, hepatic artery, and common bile duct were clipped using a microaneurysm clip (S&T AG, Switzerland) for 30 min to induce 70% hepatic ischemia, and the evidence of successful reperfusion was confirmed by the liver lobes changing from crimson to light red immediately after the occlusion. The incision was sutured after reperfusion. All the mice were sacrificed 4 h after reperfusion (Figure 1a). For blood and tissue collection, mice were anesthetized with isoflurane, and blood was collected from the inferior vena cava. The animals were then perfused with sterile saline to wash out circulating blood, followed by tissue harvesting. The selected procedures were based on established models of hepatic IRI known to reproduce clinically relevant pathological features. A murine partial hepatic ischemia model was used because it reproducibly mimics the pathophysiological features of human hepatic IRI, including oxidative stress, inflammatory responses, and hepatocellular injury. Randomization was used to allocate animals into experimental groups. Potential confounders such as cage location or measurement order were not strictly controlled; however, all animals were housed under identical environmental conditions, and procedures were conducted by the same experimenter to minimize variability. Group allocation was known to the investigator during all experimental stages, and blinding was not performed. Animals were monitored at least twice daily for signs of distress, including reduced mobility, weight loss, or abnormal behavior. Humane endpoints were predefined, and animals meeting these criteria were euthanized immediately.

### 2.3. Liver SQOR Knockdown and Hepatic IRI

**Figure 2:**
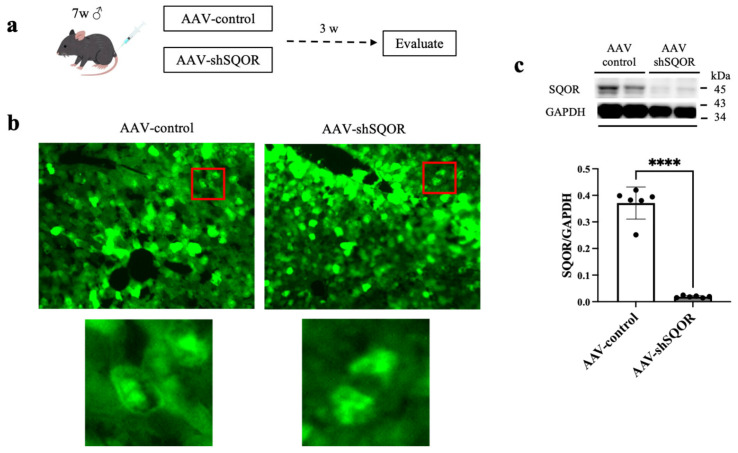
Effects of sulfide quinone oxidoreductase (SQOR) and sodium trisulfide injection on hepatic ischemia–reperfusion injury in mice. (**a**) Flowchart of injection schedule for AAV-control and AAV-shSQOR. (**b**) Representative fluorescence images of the liver sections of mice stained with an anti-GFP antibody 3 weeks after injection of AAV-control or AAV-shSQOR. The picture below is an enlarged view of the area enclosed by the red square. (**c**) Representative Western blotting images of SQOR and GAPDH protein levels and differences in SQOR/GAPDH in liver extracts from mice of AAV-control and AAV-shSQOR group. Data are expressed as the means ± standard deviations, **** *p* < 0.0001.

To investigate the role of SQOR in hepatic IRI, we used shRNA targeting mouse SQOR (shSQOR) for SQOR knockdown. For SQOR knockdown, an adeno-associated virus (AAV) vector expressing short hairpin RNA (shRNA) targeting mouse SQOR was used. The target sequence of the SQOR shRNA was 5′-GGACGTCTCTGTCAACTATAA-3′. A scrambled shRNA with no homology to known mouse genes (5′-CCTAAGGTTAAGTCGCCCTCG-3′) was used as a control. The shRNAs were packaged into ultra-purified recombinant adeno-associated virus serotype 9 (AAV9) vectors with medium-scale packaging (VectorBuilder, Chicago, IL, USA). The shSQOR vector used was pAAV [shRNA]-EGFP:T2A:Puro-U6>mSqor[shRNA#1] (Vector ID: VB221222-1295vcw), and the control vector was pAAV [shRNA]-EGFP:T2A:Puro-U6>Scramble[shRNA#1] (Vector ID: VB221222-1300gjj). AAV9 exhibits strong liver tropism following systemic administration. In mice at 7 weeks of age, adeno-associated virus vectors shSQOR (AAV-shSQOR group, n = 6) or control (AAV-control group, n = 6) were administered via the tail vein at 2.0 × 10^11^ viral particles per mouse. Hepatic IRI was induced (Figure 2a). AAV-mediated gene delivery, the vectors co-expressed green fluorescent protein (GFP) as a reporter. To verify successful AAV-mediated gene delivery, GFP expression was evaluated in frozen liver sections. Liver tissues were harvested, embedded in OCT compound, snap-frozen, and stored at −80 °C until sectioning. Cryosections (10 µm) were cut using a cryostat at −20 °C and mounted on glass slides. GFP fluorescence was directly observed using a fluorescence microscope (BZ-X800, KEYENCE, Osaka, Japan).

### 2.4. Liver SQOR Knockdown and Hepatic IRI Following Na_2_S_3_ or GSSSG Administration

To investigate the effects of supersulfides, another group of mice (AAV-shSQOR+Na_2_S_3_ group, n = 6) was treated with the hydrotrisulfide donor Na_2_S_3_ (Dojindo Laboratories, Kumamoto, Japan) via intraperitoneal injection (20 mg/kg) daily for 1 week prior to the induction of hepatic IRI. Na_2_S_3_ was freshly prepared by dissolving the compound in sterile saline immediately before use. To minimize spontaneous redox reactions and degradation into other reactive sulfur species, the solution was prepared just prior to administration, protected from light, and used within a short time frame without storage. To further validate the protective effects of supersulfides, another group of mice (AAV-shSQOR+GSSSG group, n = 6) was treated with a single, but not repeated, administration of GSSSG via intraperitoneal injection (20 mg/kg) immediately before the induction of hepatic IRI. GSSSG was prepared immediately before use. The compound was maintained as an acidic stock solution and adjusted to neutral pH when prior to administration. The solution was used without storage to minimize spontaneous redox reactions and ensure compound integrity. We synthesized GSSSG as previously described [5]. The timing of AAV-shSQOR administration and Na_2_S_3_ treatment was determined according to previous reports and preliminary experiments to ensure effective gene knockdown and adequate sulfane sulfur availability.

### 2.5. Biochemical Measurements

Before the animals were sacrificed, blood was collected from the inferior vena cava and centrifuged at 3000× *g* for 15 min to obtain the serum. Serum levels of aspartate aminotransferase (ASTT) and alanine aminotransferase (ALT) were measured using a Fuji-Drychem NX500V chemical analyzer (Fuji Film, Tokyo, Japan).

### 2.6. Measurement of the Oxidized Nicotinamide Adenine Dinucleotide (NAD^+^)/Reduced NAD (NADH) Ratio

Tissue was homogenized on ice and subjected to differential centrifugation to obtain mitochondrial and cytosolic fractions. Nucleus was removed at 600× *g* for 100 min, and mitochondria were pelleted at 10,000× *g* for 10 min and washed once. The oxidized nicotinamide adenine dinucleotide (NAD^+^)/reduced NAD (NADH) ratio in the liver tissue was measured using an NAD^+^/NADH fluorometric assay kit (Abcam, Cambridge, MA, USA; ab176723), according to the manufacturer’s protocol. In brief, 25 μL of the sample, blank control, or standard were added to 25 μL of the specific control or NAD^+^ extraction solutions. After 15 min of incubation at 37 °C, another 25 μL of control solution or NADH extraction solution (to neutralize NAD^+^ extracts) was added. Finally, 75 µL of NAD^+^/NADH reaction mixture (comprising NAD^+^/NADH recycling enzyme mixture and sensor buffer, kit components) was added, and the resulting mixtures was incubated for 1 h at room temperature. Fluorescence intensities (excitation 540 nm; emission 590 nm) were measured using an Infinite M1000 microplate reader (Tecan, Zurich, Switzerland) and i-control software (Tecan). The concentration of each component was determined from the standard solutions provided in the kit, and the NAD^+^/NADH ratio was subsequently calculated. The NAD^+^/NADH assay results were obtained from three independent experiments for each sample.

### 2.7. Histopathological Analysis

Liver tissues were fixed with 10% formalin, embedded in paraffin, sliced into 5-μm-thick sections, and stained with hematoxylin and eosin (H&E) using standard methods. Stained sections were visualized using the Axio Scan Z1 and Zen systems (Carl Zeiss AG. Ltd., Thornwood, NY, USA). Pathophysiological histological changes in hepatic IRI were evaluated for each treatment based on the Suzuki score [19]. To assess neutrophil infiltration, oxidative stress, and lipid peroxidation in the liver tissues, immunohistochemical staining was performed using specific antibodies. Myeloperoxidase (MPO, AF3667, R&D Systems) and lymphocyte antigen 6G (Ly6G, MAB10371, R&D Systems) antibodies were used to detect neutrophil accumulation in the tissue sections. To evaluate oxidative stress, 4-hydroxy-2-nonenal (4HNE, MHN-020P, JaICA) and 8-hydroxy-2-deoxyguanosine (anti-8-OHdG antibody, bs-1278R, Bioss Inc.) staining was performed. Stained sections were analyzed under a light microscope, and the number of positively stained cells or staining intensity was quantified using ImageJ version 1.53t (National Institutes of Health, Bethesda, MD, USA).

### 2.8. Western Blotting Analysis

Liver tissue was collected following dissection and frozen to examine protein expression. Tissues were homogenized in Pierce RIPA Buffer (Thermo Fisher Scientific, Waltham, MA, USA) containing a complete ethylenediaminetetraacetic acid-free protease and phosphatase inhibitor cocktail (Sigma-Aldrich, St. Louis, MO, USA). Lysates were centrifuged at 14,000× *g* for 15 min at 4 °C, and the supernatant was transferred. The protein concentration was determined using a protein quantification kit (Dojindo Laboratories, Kumamoto, Japan). Equal amounts of proteins (30 µg) were separated via sodium dodecyl sulfate-polyacrylamide gel electrophoresis (FUJIFILM Wako Pure Chemical Corporation, Osaka, Japan) and transferred to a membrane using a Trans-Blot Turbo Transfer System (Bio-Rad Laboratories, Inc., Hercules, CA, USA). After transfer, the membranes were blocked with Every blocking buffer (Bio-Rad Laboratories, Inc.) and immunoblotted with one of the primary antibodies at 4 °C overnight: mouse anti-GAPDH antibody (1:1000; Cell Signaling Technology, Inc., Danvers, MA, USA, Cat# 5174), mouse anti-CSE antibody (1:1000; Cell Signaling Technology, Cat#19689), mouse anti-CBS antibody (1:1000; Cell Signaling Technology, Cat# 14782S), mouse anti-SQOR antibody (1:500; Cell Signaling Technology, Cat#17256-1-AP), mouse anti-TST antibody (1:1000; Gene Tex Company, Cat# GTX114858), and mouse anti-ETHE1 antibody (1:1000; Proteintech Group, Inc., Cat# 27786-1AP). The bound antibody signal was detected using a horseradish peroxidase-linked antibody against rabbit IgG (1:5000; VECTOR Laboratories, Newark, CA, USA, Cat# PI-1000), and chemiluminescence was visualized using an ECL Advance Kit (GE Healthcare Bioscience, Piscataway, NJ, USA). The images were digitized, and the intensity of each band was quantified by densitometric scanning using the ImageJ software version 1.53t (National Institutes of Health). We also validated the band height of all other proteins using previous reports and manufacturers’ website.

### 2.9. Statistical Analyses

The experimental unit was a single mouse. The number of animals included in each group was 6. The sample size was based on previous studies using similar liver IRI model and was considered sufficient to detect biologically meaningful differences. No a priori statistical power calculation was performed. Data are expressed as means ± standard errors of the mean. Differences among groups were evaluated using the Mann–Whitney U test or one-way analysis of variance, followed by Tukey’s multiple comparison test. All statistical analyses were performed using GraphPad Prism 10 (GraphPad Software, San Diego, CA, USA), and statistical significance was set at *p* < 0.05.

## 3. Results

All animal experiments were conducted between April 2023 and December 2025. No animals or data points were excluded from analysis, and no adverse events were observed.

### 3.1. Hepatic IRI Model

Hepatic IRI was induced by 30 min of ischemia, followed by 4 h of reperfusion (Figure 1a). Serum levels of AST (395 ± 253 vs. 1651 ± 316 IU/L, *p* < 0.01) and ALT (332 ± 225 vs. 2325 ± 610 IU/L, *p* < 0.01) were significantly higher in the IRI group compared with the sham group (Figure 1b). Histological analysis revealed a significant increase in Suzuki score (0.33 ± 0.3 vs. 1.87 ± 0.45, *p* < 0.01), indicating severe hepatic damage (Figure 1c). The levels of CBS, CSE, ETHE1, and TST in the IRI group were not significantly different from those in the sham group, whereas the level of SQOR expression was significantly lower in the IRI group than that in the sham group (Figure 1d).

### 3.2. SQOR Knockdown Exacerbated Hepatic IRI, and Na_2_S_3_ and GSSSG Administration Provided Protection Against Hepatic IRI

Three weeks after the administration of AAV-control or AAV-shSQOR, widespread GFP expression was observed throughout the liver (Figure 2b). SQOR protein expression was significantly reduced in the AAV-shSQOR groups, indicating that SQOR was knocked down (Figure 2c).

**Figure 3:**
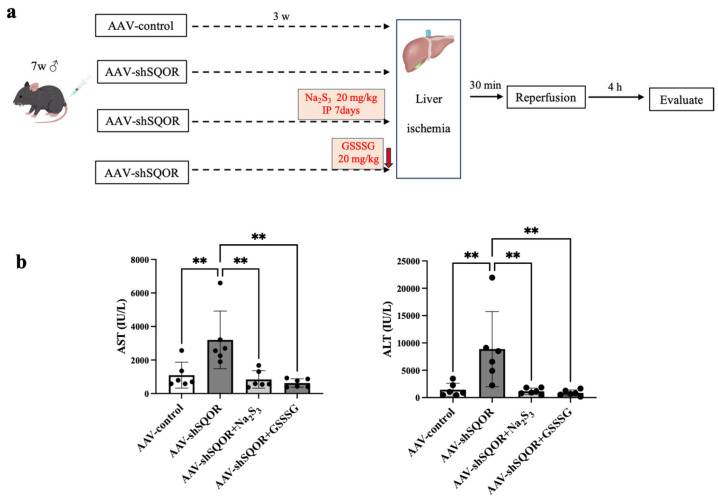
Liver function following hepatic ischemia–reperfusion injury in AAV-control, AAV-shSQOR, AAV-shSQOR+Na_2_S_3,_ and AAV-shSQOR+GSSSG mice. (**a**) Flowchart of the experimental protocols. (**b**) The levels of AST and ALT 4 h after reperfusion in mice. The data are presented as n = 6 per group. Significant differences between groups were determined using one-way analysis of variance. Data are expressed as the means ± standard deviations, ** *p* < 0.01.

Among the AAV-control, AAV-shSQOR, AAV-shSQOR+Na2S3, and AAV-shSQOR+GSSSG groups, AST and ALT levels were significantly higher in the AAV-shSQOR group than in the other groups (AST: 1096 ± 774 vs. 3201 ± 1720 vs. 840 ± 524 vs. 627 ± 252 IU/L, *p* < 0.01, ALT: 1434 ± 1191 vs. 8874 ± 6881 vs. 1159 ± 576 vs. 858 ± 532 IU/L, *p* < 0.01) (Figure 3b).

**Figure 4:**
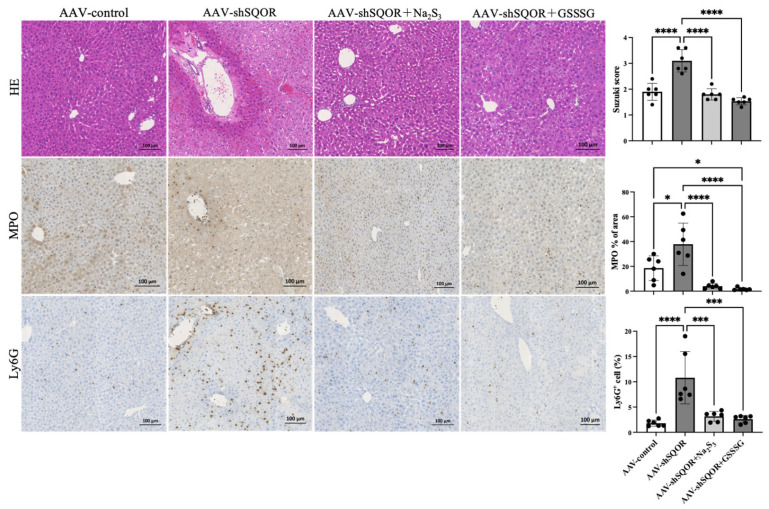
Hepatic histological changes in AAV-control, AAV-shSQOR, AAV-shSQOR+Na_2_S_3_, and AAV-shSQOR+GSSSG mice. Representative images of hematoxylin and eosin staining and immunohistochemical expression of myeloperoxidase (MPO) and lymphocyte antigen 6G (Ly6G) 4 h after reperfusion in mice from each group. The right panels show the differences in Suzuki score, MPO immunoreactivity levels, and Ly6G immunoreactivity levels in the mice of each group. The data are presented as n = 6 per group. Significant differences between groups were determined using one-way analysis of variance. Data are expressed as the means ± standard deviations, * *p* < 0.05, *** *p* < 0.001, and **** *p* < 0.0001.

Among the AAV-control, AAV-shSQOR, AAV-shSQOR+Na2S3, and AAV-shSQOR+GSSSG groups, the Suzuki score was significantly higher in AAV-shSQOR group compared with the other groups (1.9± 0.33 vs. 3.1 ± 0.43 vs. 1.8 ± 0.22 vs. 1.53 ± 0.14, *p* < 0.01) (Figure 4). Increased MPO and Ly6G reflect augmented neutrophil recruitment and activation in the liver, suggesting an enhanced inflammatory response that contributes to hepatocellular damage during hepatic IRI [19]. Immunohistochemical staining revealed that the AAV-shSQOR group exhibited significantly higher levels of MPO and Ly6G in the liver compared with the other groups. Quantitative analysis confirmed significantly larger immune-positive areas for both markers in the AAV-shSQOR group (MPO: 18.5 ± 9.9 vs. 37.8 ± 17.1 vs. 4.0 ± 2.2 vs. 1.7 ± 1.1%, *p* < 0.01, Ly6G: 1.8 ± 0.6 vs. 10.8 ± 5.2 vs. 3.2 ± 0.9 vs. 2.6 ± 0.7%, *p* < 0.01) (Figure 4).

### 3.3. SQOR Knockdown Increases Oxidative Stress, Whereas Na_2_S_3_ and GSSSG Administration Attenuates Oxidative Stress in Hepatic IRI

**Figure 5:**
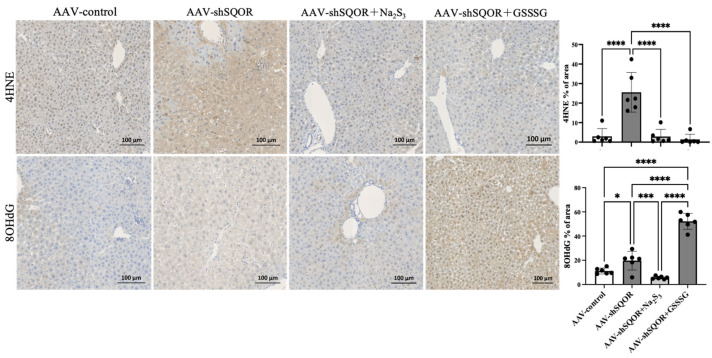
Hepatic histological changes in AAV-control, AAV-shSQOR, AAV-shSQOR+Na_2_S_3_, and AAV-shSQOR+GSSSG mice. Representative images of the immunohistochemical expression of 4-hydroxy-2-nonenal (4HNE) and 8-hydroxy-2-deoxyguanosine (8OHdG) 4 h after reperfusion in mice from each group. The right panels show differences in 4HNE and 8OHdG immunoreactivity levels in mice of each group. The data are presented as n = 6 per group. Significant differences between groups were determined using one-way analysis of variance. Data are expressed as the means ± standard deviations, * *p* < 0.05, *** *p* < 0.001, and **** *p* < 0.0001.

Hepatic IRI is characterized by abrupt reoxygenation, which lead to excessive generation of reactive oxygen species (ROS) from mitochondrial dysfunction and activated inflammatory cells [20,21]. 4HNE is a major end product of lipid-peroxidation induced by ROS and reflects oxidative damage to cellular and mitochondrial membranes during IRI [22]. 8OHdG is generated by oxidtive modification of DNA bases and serves as an indicator of oxidative DNA damage following ischemic reperfusion [23]. The accumulation of 4HNE and 8OHdG is therefore of particular significance in hepatic IRI. Among the AAV-control, AAV-shSQOR, AAV-shSQOR+Na2S3, and AAV-shSQOR+GSSSG groups, immunohistochemical staining revealed that the AAV-shSQOR group exhibited significantly higher levels of 4HNE in the liver compared with the other groups, and the AAV-shSQOR group exhibited significantly higher levels of 8OHdG in the liver compared with the AAV-control and AAV-shSQOR+Na_2_S_3_ groups, and there was significant increase of 8OHdG in the AAV-shSQOR+GSSSG group. Quantitative analysis confirmed significantly larger immune-positive areas for both markers. (4HNE: 2.9 ± 4.0 vs. 25.5 ± 10.2 vs. 2.9 ± 3.7 vs. 1.4 ± 2.6%, *p* < 0.01, 8OHdG: 11.2 ± 2.5 vs. 19.7 ± 7.7 vs. 5.6 ± 1.2 vs. 52.2 ± 6.6%, *p* < 0.01) (Figure 5).

### 3.4. Cellular Metabolism in Hepatic IRI

**Figure 6:**
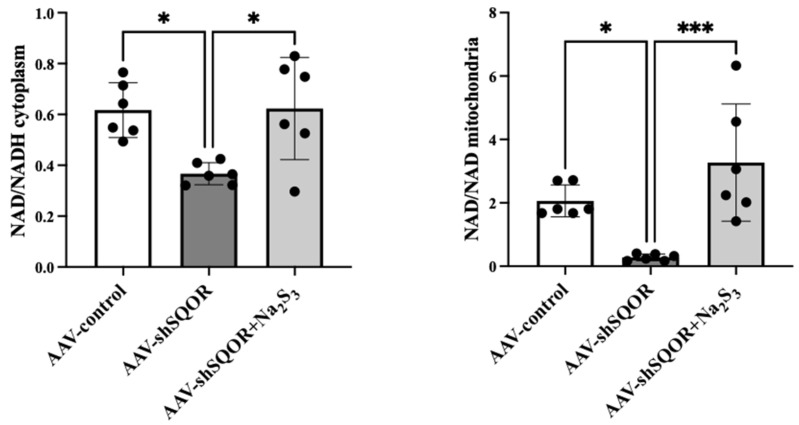
Cellular redox balance in AAV-control and AAV-shSQOR and AAV-shSQOR+Na_2_S_3_ mice. Levels of the oxidized nicotinamide adenine dinucleotide (NAD^+^)/reduced NAD (NADH) ratio in the cytoplasm and mitochondria 4 h after reperfusion in mice. Data are expressed as the means ± standard deviations, * *p* < 0.05, and *** *p* < 0.001.

During hepatic IRI, impaired electron transport leads to NADH accumulation and reduced NAD^+^/NADH ratio, which has been widely used as an indicator of mitochondrial redox imblance [24]. Among the AAV-control, AAV-shSQOR, and AAV-shSQOR+Na2S3, the cytoplasm and mitochondria NAD^+^/NADH ratio in the liver of AAV-shSQOR group were significantly lower than those in the other groups (NAD^+^/NADH in the cytoplasm: 0.62 ± 0.11 vs. 0.37 ± 0.04 vs. 0.62 ± 0.2, *p* < 0.01, NAD^+^/NADH in the mitochondria: 2.06 ± 0.50 vs. 0.28 ± 0.10 vs. 3.27 ± 1.85, *p* < 0.01) (Figure 6).

### 3.5. Levels of CBS, CSE, ETHE1, and TST in Hepatic IRI Under SQOR Knockdown and Na_2_S_3_ Administration

**Figure 7:**
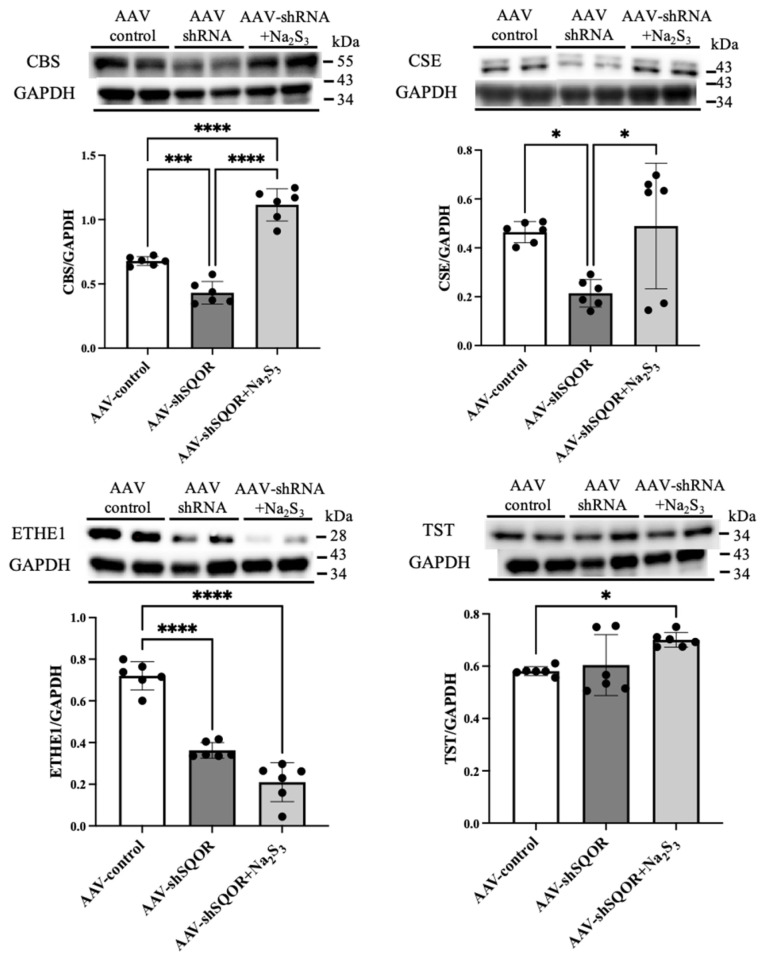
Relative protein levels of cystathionine β-synthase (CBS), cystathionine γ-lyase (CSE), ethylmalonic encephalopathy 1 (ETHE1), and thiosulfate sulfurtransferase (TST) in the livers of AAV-control and AAV-shSQOR and AAV-shSQOR+Na_2_S_3_ mice. Representative Western blotting images of CBS, CSE, ETHE1, TST, and GAPDH protein levels in liver extracts from each group of mice and differences in CBS/GAPDH, CSE/GAPDH, ETHE1/GAPDH, and TST/GAPDH in each group. The data are presented as n = 6 per group. Significant differences between groups were determined using one-way analysis of variance. Data are expressed as the means ± standard deviations, * *p* < 0.05, *** *p* < 0.001, and **** *p* < 0.0001.

Among the AAV-control and AAV-shSQOR and AAV-shSQOR+Na_2_S_3_ groups, the levels of CBS and CSE expression were significantly lower in the AAV-shSQOR group compared with those in the AAV-control group, whereas the levels of CBS and CSE were significantly higher in the AAV-shSQOR+Na_2_S_3_ group compared to those in the AAV-control group (CBS: 0.68 ± 0.03 vs. 0.43 ± 0.09 vs. 1.11 ± 0.13, *p* < 0.01, CSE: 0.46 ± 0.04 vs. 0.21 ± 0.06 vs. 0.49 ± 0.26, *p* < 0.01) (Figure 7). The level of ETHE1 expression was lower in the AAV-shSQOR group than in the AAV-control group, and it was even lower in the AAV-shSQOR + Na_2_S_3_ group compared with the AAV-shSQOR group (0.72 ± 0.07 vs. 0.36 ± 0.04 vs. 0.21 ± 0.09, *p* < 0.01) (Figure 7). The level of TST expression was higher in the AAV-shSQOR+Na_2_S_3_ group than in the AAV-control group (0.58 ± 0.02 vs. 0.60 ± 0.12 vs. 0.70 ± 0.03, *p* < 0.01) (Figure 7).

## 4. Discussion

This study revealed that SQOR is required for the protective response against hepatic IRI. Furthermore, the supersulfides products produced by SQOR may reduce hepatic IRI. To the best of our knowledge, no study has demonstrated inhibitory effects of SQOR and supersulfides on hepatic IRI. This is a novel finding regarding the effects of sulfide pathways on hepatic IRI.

In hepatic IRI, the expression of SQOR was lower compared to that in the sham group, whereas the expression of other key enzymes involved in sulfur metabolism, such as CBS, CSE, ETHE1, and TST, remained unchanged. Notably, the expression of TST, another mitochondrial protein, was not altered after IRI, suggesting that SQOR downregulation may not solely reflect a general reduction in mitochondrial abundance. This selective downregulation of SQOR suggests that SQOR may play a critical or rate-limiting role in the regulation of mitochondrial sulfur metabolism during hepatic IRI.

First, we tried using an AAV-encoding SQOR (AAV-SQOR) to express SQOR specifically in the liver of mice but we did not find increase in SQOR. AAV-SQOR-injected mice reportedly had higher levels of SQOR in the brain [9]. We hypothesized that the liver has a higher basal expression level of SQOR than the brain, which could limit the increase in protein levels, even after the injection of AAV-SQOR. Because SQOR is localized in the inner mitochondrial membrane [9,25] and there is inherently high basal expression of SQOR in the liver [26], the mitochondrial translocation system may be functionally saturated, restricting the import of exogenously delivered proteins [27]. Therefore, we conducted the experiments using AAV-shSQOR.

SQOR is a key enzyme in the supersulfides oxidation pathway [28] and the rate-limiting enzyme in sulfide oxidation, which is a critical initial step [25,29]. During ischemic hypoxia, electrons are absorbed by sulfur and sulfur compounds, such as hydrogen disulfide (HSSH) and hydrogen trisulfide, and SQOR facilitates electron transfer from these sulfur compounds to the mitochondrial electron transport chain [30]. In this study, SQOR silencing suppressed electron transport to the mitochondrial electron transport chain, leading to a decreased NAD^+^/NADH ratio in the AAV-shSQOR group. In contrast, in the AAV-shSQOR+Na_2_S_3_ group, the administration of Na_2_S_3_ improved the NAD^+^/NADH ratio because of enhanced resistance to oxidative stress induced by Na_2_S_3_, which may have suppressed reactive oxygen species [31] production during ischemia, thereby indirectly increasing the NAD^+^/NADH ratio. Although a few studies have examined the role of SQOR in hepatic ischemia, our study is the first to clarify the role of SQOR in hepatic IRI.

We hypothesized that SQOR is related to supersulfides production, which may enhance the resistance to oxidative stress induced by hepatic IRI. Firstly, we investigated the effect of short-term repeated dosing of Na_2_S_3_. Following administration, Na_2_S_3_ is rapidly redistributed in vivo into sulfide–persulfide pools. These persulfide species emerge within minutes after administration and exert biological effects on a timescale of minutes to hours. However, in the present study, repeated administration of Na_2_S_3_ was employed to modify the basal redox state prior to ischemia, thereby maintaining an elevated basal persulfide tone and enhancing mitochondrial sulfide-handling capacity in a preconditioning-like manner. In the AAV-shSQOR+Na_2_S_3_ group, the AST and ALT levels and pathological findings were similar to those in the AAV-control group, while the protein levels of CBS and CSE were similarly maintained. This suggests that the maintenance of supersulfides by CBS and CSE or repeated injection of Na_2_S_3_ may have a protective effect against hepatic IRI. Moreover, to confirm the direct impact of supersulfides, rather than the secondary effects of supersulfide treatment, such as transcriptional modulation caused by repeated administration, we evaluated the effect of GSSSG administered alone just before hepatic IRI. GSSSG, as a glutathione-derived persulfide, is structurally distinct from Na_2_S_3_ and is expected to exhibit greater chemical stability and intracellular persistence [Supplementary Figure S1]. Based on these properties, we hypothesized that GSSSG could exert biological effects during the early reperfusion phase even after a single administration. In the AAV-shSQOR+GSSSG groups, the AST and ALT levels were similar to those in the AAV-control group, suggesting that supersulfides may have a protective effect against hepatic IRI. However, the most interesting point is that while the accumulation of 4HNE was reduced by GSSSG administration, the accumulation of 8OHdG increased further upon GSSSG administration. We consider that this phenomenon was related to a bias in the site of action based on the molecular properties of GSSSG. GSSSG tend to localize near cell membranes, protein thiols, and the vicinity of mitochondria [10], exhibiting particularly potent antioxidant effects against the elimination of lipid radicals [11]. It may be interpreted that GSSSG shifted the disease pattern towards more reparable DNA oxidative damage by suppressing immediate and lethal lipid peroxidation. Given that DNA oxidative damage can be addressed by repair mechanisms such as base excision repair, this change does not necessarily signify worsening of the impairment.

GSSSG is a component of endogenous trisulfide [12] and thus we expect a similar protective effect of exogenously administered GSSSG to that of endogenous GSSSG. GSSSG is also more stable than Na_2_S_3_ in physiological fluids and has been proven to increase S0 in the CNS [13,14]. Previous studies have shown that supersulfides-containing species, such as GSSH, HSSH, and CysSSH, exhibit strong scavenging activities for hydrogen peroxide and electrophiles, which contribute to the regulation of redox signaling [31]. These findings indicate that supersulfides has potential as a novel therapeutic agent for the prevention of hepatic IRI, particularly in clinical settings, such as in cases of liver transplantation and ALI caused by various shocks.

CSE and CBS play essential roles in sulfide production. CSE induction by stressors, especially those associated with increased ROS formation, can be considered a homeostatic response, as it facilitates the synthesis of antioxidant molecules, including cysteine, GSH, and supersulfides, restoring the redox balance [32]. CBS catalyzes multiple reactions using substrates, such as serine, cysteine, and homocysteine [30,33]. A novel finding of this study was that SQOR silencing reduced the protein levels of CBS and CSE in hepatic IRI, whereas these protein levels were maintained by administering Na_2_S_3_. Severe or sustained oxidative stress and the accumulation of ROS suppress the expression of CSE and CBS [34,35]. In this study, severe or sustained oxidative stress and ROS accumulation may have led to the suppression of CSE and CBS expression in the AAV-shSQOR group. In contrast, our model represents an early reperfusion phase with moderate injury severity, in the AAV-shSQOR+Na_2_S_3_ group, the expression of CSE and CBS was preserved, which suggests that Na_2_S_3_ administration alleviates oxidative stress and ROS accumulation and that the expression of CSE and CBS may have been maintained.

ETHE1 and TST, such as SQOR, are key enzymes in supersulfides oxidation pathways [36]. Sulfide is oxidized by the SQOR to supersulfides, which ETHE1 subsequently oxidizes to sulfite, and TST further converts sulfite to sulfate or thiosulfate [29,37]. In this study, the protein expression of ETHE1 was significantly decreased in the AAV-shSQOR group. Silencing of SQOR can reduce the production of supersulfides, such as GSSH, which function as substrates for ETHE1 [29,37,38]. Depletion of these substrates may decrease the demand for ETHE1 activity, potentially leading to the feedback-dependent downregulation of ETHE1 expression [37]. In contrast, the observed maintenance of the protein expression of TST may be attributed to a reduction in its substrate availability, resulting from the decreased expression of ETHE1. This could be because TST is located downstream of the supersulfides oxidation pathways [37,39]. Diminished upstream production of substrates may reduce the demand for TST activity, thereby stabilizing its expression. These results suggest that silencing SQOR can alter the expression of downstream enzymes, such as ETHE1 and TST, through both substrate availability and feedback mechanisms.

A limitation of our study is that it focused exclusively on pathways related to supersulfides production and oxidation, without investigating other regulatory pathways, such as hypoxia-inducible factor-1 and nuclear factor kappa B pathways. Most importantly, we did not directly measure the decrease in supersulfides levels in shSQOR. However, persulfides are reportedly produced as reaction products of SQOR [29,30,37], and a decrease in persulfides levels has been observed in SQOR-deficient mice [40]. Therefore, it is reasonable to expect that a decrease in shSQOR would also lead to a decrease in supersulfides. Further studies on this topic are required. Secondly, we focused on only male that are more sensitive to ischemia than female, which we believe augments the significance of our current study not only in mice but also in human, considering that male patients tend to experience more severe IRI than do female patients clinically [41,42]. Therefore, the present findings may not be directly extrapolated to female mice, and potential sex-specific differences warrant further investigation. Third, the effects of exogenous supersulfides in the absence of SQOR knockdown mice were not examined. The present experiments were designed to specifically evaluate SQOR-dependent mechanisms under moderate hepatic IRI. Evaluation of exogenous supersulfides in normal conditions may require a more severe injury model to adequately reveal their protective effects, and this warrants further investigation. Forth, the effects of Na_2_S_3_ and GSSSG were evaluated at a single dose of 20 mg/kg. Dose–response relationships, including the effects of higher or lower doses were not examined. Therefore, the optimal dosing range and the extent to which dose escalation might further enhance or alter the observed protective effects remain to be determined in future studies.

Although this study was conducted in a murine model of acute hepatic IRI, the role of SQOR in mitochondrial sulfide oxidation and redox homeostasis is highly conserved across mammalian species. Therefore, the fundamental mechanisms identified in this study are likely to be relevant to other species and experimental settings involving ischemic or hypoxic stress. However, differences in metabolism, disease context, and the acute nature of this model may limit direct extrapolation to chronic liver diseases or clinical settings. Nevertheless, our findings provide mechanistic insights that may be relevant to human hepatic IRI, such as that occurring during liver transplantation or major hepatic surgery.

## 5. Conclusions

SQOR may be essential for protective response against hepatic IRI in mice. Furthermore, the administration of the exogenous supersulfide donor maintains the liver supersulfide level and protects mice against hepatic IRI.

## Data Availability

The original contributions presented in this study are included in the article. Further inquiries can be directed to the corresponding author.

## Supplementary Materials

The following supporting information can be downloaded at: https://www.mdpi.com/article/10.3390/antiox15010094/s1, Figure S1: Time-dependent changes in plasma sulfur metabolic profiles following GSSSG ip administration.

## Abbreviations

ALI	acute liver injury
BUN	blood urea nitrogen
CBS	cystathionine β-synthase
CSE	cystathionine γ-lyase
CysSSH	cysteine persulfides
ETHE1	ethylmalonic encephalopathy1
GSSSG	glutathione trisulfide
GSSH	glutathione persulfides
H_2_S	hydrogen sulfide
HSSH	hydrogen disulfide
IRI	ischemia–reperfusion injury
PBS	phosphate-buffered saline
ROS	reactive oxygen species
SQOR	sulfide quinone oxidoreductase
TST	thiosulfate sulfurtransferase
