# Supplementary figures and images for: Role of Sulfide Quinone Oxidoreductase and Supersulfides in Hepatic Ischemia–Reperfusion Injury in Mice
Source: Antioxidants (Basel). 2026 Jan 12;15(1):94. doi: 10.3390/antiox15010094 (PMC12838252; doi:10.3390/antiox15010094)

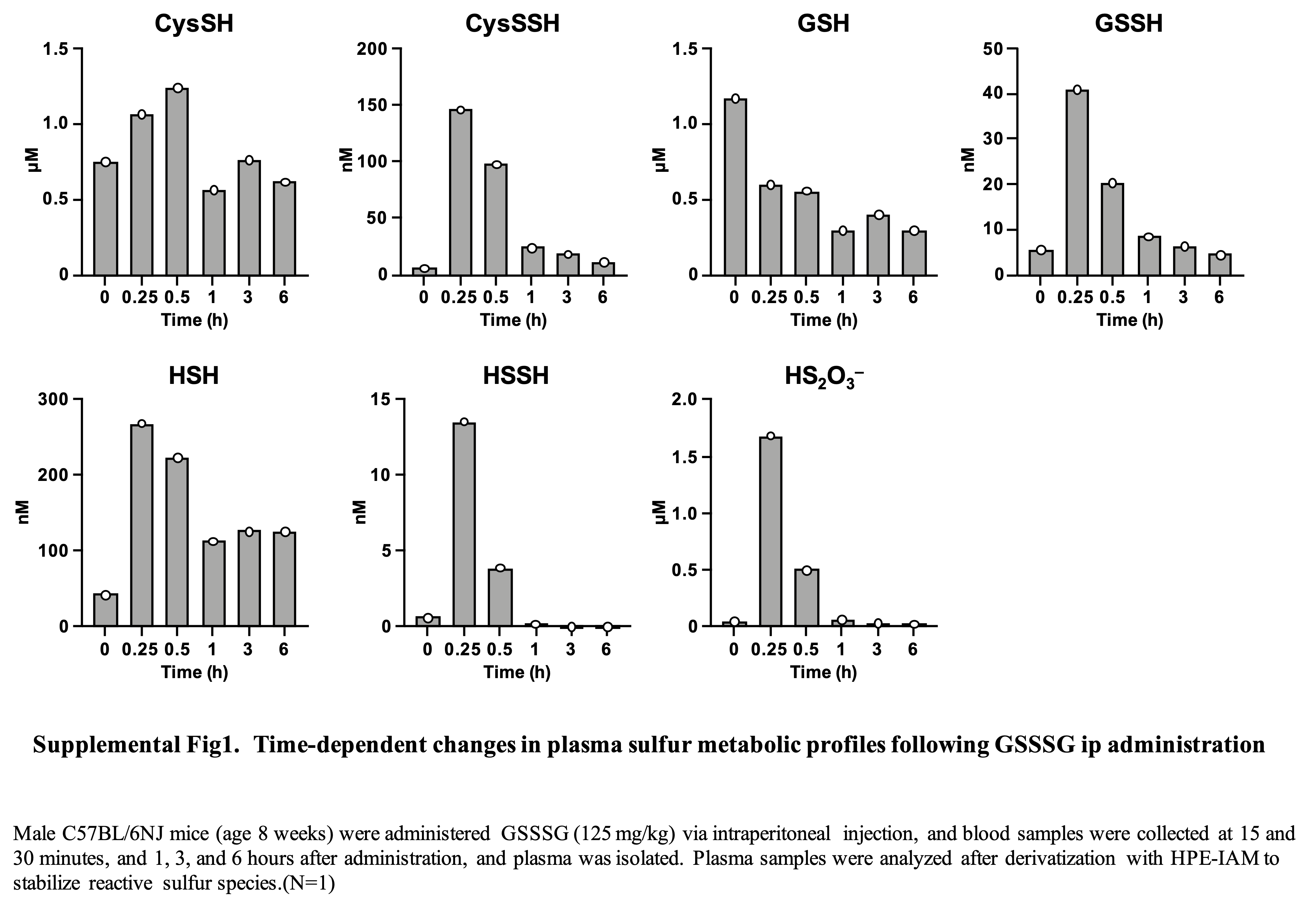

Supplement: Supplementary file 1 [file antioxidants-15-00094-s001.zip › antioxidants-4065669-supplementary.tiff]
